# MicroRNA-195-5p, a new regulator of Fra-1, suppresses the migration and invasion of prostate cancer cells

**DOI:** 10.1186/s12967-015-0650-6

**Published:** 2015-09-04

**Authors:** Jian Wu, Alin Ji, Xiao Wang, Yi Zhu, Yasai Yu, Yiwei Lin, Yunfu Liu, Shiqi Li, Zhen Liang, Xin Xu, Xiangyi Zheng, Liping Xie

**Affiliations:** Department of Urology, The First Affiliated Hospital, School of Medicine, Zhejiang University, Qingchun Road 79, Hangzhou, 310003 Zhejiang China

**Keywords:** miR-195, Fra-1, Prostate cancer, Migration, Invasion

## Abstract

**Background:**

An increasing number of studies have demonstrated that deregulation of microRNAs (miRNAs) was a common event in tumor tissues and miRNAs would be treated as ideal tumor biomarkers or therapeutic targets. miR-195-5p (termed as miR-195 for briefly in the following part) was suggested to function as a tumor suppressor in cancer development and progression. However, the roles of miR-195 in human prostate cancer are still elusive. Thus, this study was performed to investigate the biological functions and its molecular mechanisms of miR-195 in human prostate cancer cell lines, discussing whether it has a potential to be a therapeutic way of prostate cancer.

**Methods:**

Two human prostate cancer cell lines were analyzed for the expression of miR-195 by quantitative real-time reverse transcription–polymerase chain reaction (RT–PCR). A gain-of-function study of miR-195 was conducted by transfecting mimics into DU145 and PC3 cells and cell motility and invasion ability were evaluated by wound healing assay and transwell assay. Tissue microarray, and immunohistochemistry with antibodies against Fra-1 was performed using the peroxidase and DAB methods. The target gene of miR-195 was determined by luciferase assay, quantitative RT–PCR and western blot. The regulation of motility by miR-195 was analyzed by western blot.

**Results:**

miR-195 was frequently down-regulated in both prostate cancer cell lines, DU145 and PC3. Overexpression of miR-195 significantly repressed the capability of migration and invasion of prostate cancer cells. In addition, we identified Fra-1, a cell motility regulator, as a novel target of miR-195. Fra-1 was up-regulated in prostate cancer tissues. We also observed that inhibition of miR-195 or restoration of Fra-1 in miR-195-over-expressed prostate cancer cells partially reversed the suppressive effects of miR-195. Furthermore, we demonstrated miR-195 could inhibit prostate cancer cell motility by regulated the expression of c-Met, MMP1, MMP9.

**Conclusions:**

miR-195 can repress the migration and invasion of prostate cancer cells via regulating Fra-1. Our results indicate that miR-195 could be a tumor suppressor and may have a potential to be a diagnostics or therapeutic target in prostate cancer.

**Electronic supplementary material:**

The online version of this article (doi:10.1186/s12967-015-0650-6) contains supplementary material, which is available to authorized users.

## Background

Prostate cancer is the most frequently diagnosed male cancer and the second-leading cause of oncological mortality in men living in the United States [1]. It is a clinically heterogeneous, multifactorial disease and the incidence is continuously rising. Like many other solid tumors, prostate cancer mortality attributes largely to cancer metastasis, a complicated process that involves changes in the extracellular matrix to support invasion, increased cell motility and the ability of cells to initiate and maintain growth at a distant site [2]. However, unlike a majority of solid cancers, prostate cancer usually shows poor response to chemotherapy. Therefore, more effective strategies for the targeted treatment of prostate cancer, especially for cancer metastasis, are urgently needed.

Carcinogenesis and progression are multistep processes, involving numerous genetic mutations, aberrant gene expression, and microRNA (miRNA) dysregulation [3]. MicroRNAs (miRNAs), which are widespread in eukaryotic cells, are endogenous single stranded, non-protein-coding RNAs of approximately 22 nucleotides in length. Numerous cellular processes are affected by miRNA, including differentiation, proliferation, cell-cycle control, apoptosis, migration and invasion [4]. miRNAs exert their regulatory effects by binding to partially complementary sequences in the 3′-untranslated region (3′UTR) of target mRNAs, reducing the stability and/or translation efficiency of target mRNAs in a sequence-specific manner. Accumulating evidence suggests that miRNAs are significant molecules in diverse cancers, including prostate cancer, by regulating the expression of various oncogenes and tumor suppressors [5, 6]. Large scale miRNA expression analyses indicated a common deregulation of miRNAs in tumors compared to their benign counterparts. For example, in the prostate cancer miRNA signature analysis carried out by Porkka et al. [7], expression profiling of 319 miRNAs in 6 prostate cancer cell lines, 9 prostate cancer xenografts samples, and 13 clinical prostate tissue samples were used to classify prostate tumors. Here, 37 miRNAs (miR-16, miR-23a, miR-23b, miR-143, miR-145, miR-195,miR-221, miR-222, miR-497 et al.) were found to be downregulated in hormone-refractory late-stage prostate carcinomas, whereas 14 miRNAs were upregulated in hormonerefractory carcinomas. Schaefer et al. [8] validated that ten microRNAs (hsa-miR-16, hsa-miR-31, hsa-miR-125b, hsa-miR-145, hsa-miR-149, hsa-miR-181b, hsa-miR-184, hsa-miR-205, hsa-miR-221, hsa-miR-222) were downregulated and five miRNAs (hsa-miR-96, hsa-miR-182, hsa-miR-182, hsa-miR-183, hsa-375) were upregulated in prostate cancer. Meanwhile, several miRNAs were also shown to be deregulated and functionally relevant across different cancer diseases: down-regulation of miR-125a and miR-125b in breast cancer [9], the let-7 miRNAs in lung cancer [10], and miR-143 and miR-145 in different cancer types [11]. Besides, further investigations have assessed the diagnostic and prognostic value of miRNA profiling in prostate cancer. Of note, two promising miRNAs, miR-141 and miR-375, were suggested as diagnostic and prognostic marker across independent studies. Both miRNAs were found to be elevated in serum of men with metastatic castration-resistant prostate cancer compared to healthy individuals [12, 13]. To elucidate the role of the individual miRNAs in prostate cancer, genes and pathways which are regulated by the differentially expressed miRNAs should be studied. Thus, a set of targets of miRNAs have been identified in relevant studies. The restoration of miR-205 has been shown to restrain cell viability via targeting MED1, miR-124 targets androgen receptor and inhibits proliferation of prostate cancer cells, miR -23b represses proto-oncogene Src kinase, miR-125a and miR-125b suppress the oncogenes ERBB2 and ERBB3 [14–17]. Although the study of miRNAs is still challenging due to variable downstream regulators, the above evidence gives us a promising outlook for the application of miRNAs for prostate cancer management.

Several recent studies have presented a global analysis of miR-195 expression and function in different human cancers. Our previous study showed that overexpression of miR-195 could induce G1-phase arrest by targeting the novel target CDK4 in bladder cancer cells [18]. It was reported that miR-195 could block the G(1)/S transition in hepatocellular carcinoma cells by repressing Rb-E2F signaling through targeting multiple molecules, including cyclin D1, CDK6, and E2F3 [19]. miR-195 played a tumor-suppressor role in human glioblastoma cells by targeting E2F3 and CCND3 involved in cellular proliferation and invasion [20]. Besides, it also promoted colorectal cancer cell apoptosis by repressing Bcl-2 [21]. In the current study, we aimed to determine the role of miR-195 in determining the aggressiveness of prostate cancer cells and studied the correlated regulatory mechanism of miR-195. We found that miR-195 expression was downregulated in two prostate cancer cell lines, ectopic expression of miR-195 significantly suppressed cell migratory and invasive capacities of PC3 and DU145 cells through inhibition of Fra-1. These findings indicated that miR-195 could be a potential tumor suppressor by directly binding to Fra-1 in prostate cancer.

## Methods

### Cell lines and cell culture

The human prostate cancer cell lines PC3, Du145 and normal human prostate cells (RWPE-1) were obtained from the Shanghai Institute of Cell Biology (Shanghai, China), and were cultured in RPMI 1640 medium supplemented with 10 % heat-inactivated fetal bovine serum and incubated at 37 °C in a humidified incubator with 5 % CO_2_.

### miRNAs and transfection

miR-195 mimic(sense 5′-UAGCAGCACAGAAAUAUUGGC-3′) and the negative control(sense 5′-ACUACUGAGUGACAGUAGA-3′) were chemically synthesized by GenePharma (Shanghai, China). For convenience, miR-195 mimic and the negative control are termed miR-195 and NC, respectively. The day before transfection, the cells were plated to 60–70 % confluency in growth medium without antibiotics. The transfection was performed using Lipofectamine 2000 Reagent (Invitrogen, Carlsbad, CA, USA) according to the manufacturer’s instruction.

### RNA extraction and real-time quantitative PCR (qPCR)

Total RNA was extracted from cultured cells by using TRIzol reagent (Takara, Japan) and reverse transcribed into cDNA using a PrimeScript^®^ RT reagent Kit (Takara, Japan), while microRNA was isolated with RNAiso for Small RNA (Takara, Japan). Before PCR reaction, miRNA was polyadenylated and reverse transcribed using a One Step PrimeScript^®^ miRNA cDNA Synthesis Kit (Takara, Japan). The levels of mRNAs and miRNAs were measured by real-time quantitative *PCR* using an ABI 7500 Real-Time PCR System (Applied Biosystems, Carlsbad, USA) with SYBR Premix Ex Taq II (TaKaRa, Japan). Glyceraldehyde-3-phosphate dehydrogenase (GAPDH) and U6 small nuclear RNA were used as internal controls for detection. The relative expression level of miR-195 and Fra-1 was calculated and quantified with the 2^−ΔΔCt^ method after normalization. All the primer sequences (forward and reverse) are listed as follows:

(1) miR-195: GATAGCAGCACAGAAATATTGGC; (2) U6: TGCGGGTGCTCGCTTCGGCAGC; (3) GAPDH F: AAGGTGAAGGTCGGAGTCA and GAPDH R: GGAAGATGGTGATGGGATTT; (4) Fra-1 F: CAGCTCATCGCAAGAGTAGCA and Fra-1 R: CAAAGCGAGGAGGGTTGGA.

### Luciferase activity assay

We designed oligonucleotide pairs that contain the regions with or without a possible binding site from the 3′ untranslated region (UTR) of Fra-1,then the desired sequences were annealed and ligated into the pmirGLO Dual-Luciferase miRNA Target Expression Vector (Promega, USA) between the *Sac*I and *Sal*I sites according to the manufacturer’s protocol. Both insertions were verified by sequencing. HEK293T cells were co-transfected with 0.2 µg luciferase reporter vector comprising wildtype or mutant 3′-UTR and 50 nM miR-195 or miR-NC, using Lipofectamine 2000 (Invitrogen) in the 24-well plates. The cells were harvested 48 h post-transfection, and the luciferase activity was measured by Dual-Luciferase Reporter Assay System (Promega, USA). Luciferase activities were shown as firefly luciferase/renilla luciferase ratio.

### Cell proliferation assay

Cells were seeded in 96-well plate at a density of 4 × 10^3^/well. After an overnight incubation, the cells were transfected with the RNA duplexes (miR-195, siFra-1or control) for 2–3 days. Subsequently, the medium was removed, cell counting solution (WST-8, Dojindo Laboratories, Japan) was added to each well, and the cells incubated for an additional 1 h. The absorbance of the solution was measured spectrophotometrically at 450 nm with a MRX II absorbance reader (Dynex Technologies, USA).

### Cell migration and invasion assay

In vitro, cell migration and invasion assays were performed using transwell chambers (pore size of 8 µm; Costar, Corning, Switzerland), which bottom was coated with 1 mg/ml BD Matrigel Matrix (BD Biosciences, USA) for invasion assays specifically. The inserts were placed in the 24-well culture plates. PC3 and Du145 cells were transfected as mentioned above. Post-transfected cells (48 h) were trypsinized and resuspended in serum-free RPMI 1640 medium, and 200 µl of the cell suspension (8–10 × 10^4^ cells) was added to the upper chamber of uncoated (for migration assays) or Matrigel coated (for invasion assays) membranes, meanwhile 600 µl of medium containing 10 % fetal bovine serum was added to the lower chamber as chemoattractant. After 24 or 48 h of incubation at 37 °C in a 5 % CO_2_ humidified atmosphere, the cells that had not migrated through the pores were manually removed from the upper face of the filters using cotton swabs, and Cells adherent to the bottom surface of the inserts were fixed in cold 100 % methanol for 10 min and then stained with 0.01 % crystal violet for 2 min. Finally the filters were washed thoroughly in water and images were taken under a microscope (Olympus, Japan) with appropriate magnification. These experiments were done in triplicate and performed a minimum of three times.

### Wound healing assay

Approximately 1 × 10^5^ PC3 or Du145 cells were plated in each well of a 6-well plate. After overnight incubation, cells were respectively transfected with 50 nM miR-195 or miR-NC. When cell confluence reached about 90–100 % after transfection, wounds were created in confluent cells using a 200 µl pipette tip. The cells were rinsed several times with media to remove any free-floating cells and debris, and placed back in growth medium. Wound healing was observed at different time points(0, 24, 48 h)within the scrape line, and representative scrape lines for each cell type were photographed using phase-contrast microscope (Olympus, Japan). The results were quantified by Image J software.

### Western blotting analysis

In brief, the transfected cells were harvested, washed and lysed with RIPA buffer. The protein concentration was measured using the bicinchoninic acid protein assay kit (Pierce Biotechnology, Rockford, IL, USA). Equivalent quantities (30–50 µg) of protein were separated by 12 % sodium dodecyl sulfate polyacrylamide gel electrophoresis (SDS-PAGE) and transferred to polyvinylidene fluoride microporous (PVDF) membranes (Millipore, Bedford, MA, USA). The membranes were blocked with 5 % non-fat milk in Tris-buffered saline for 2 h and incubated overnight at 4 °C with primary antibodies against Fra-1, MMP9, c-Met, GAPDH, HMGA1, vimentin antibody (Epitomics, Burlingame, USA), MMP1 (Santa Cruz) at dilutions specified by the manufacturer. The membranes were washed 3 times in TBS-Tween and incubated with the corresponding horseradish peroxidase (HRP)-conjugated secondary antibody at a 1:5000 dilution for 1 h. Bound secondary antibody was detected using an enhanced chemiluminescence (ECL) system (Pierce Biotechnology Inc, Rockford, USA). Western-blot results were analyzed quantitatively by Image J Software.

### Fra-1 suppression by siRNA

Small silencing RNA (siRNA) interfering Fra-1 (siFra-1) (sense 5′-CACCAUGAGUGGCAGUCAGdTdT-3′ and antisense 5′-CUGACUGCCACUCAUGGUGdTdT-3′) and the negative control siRNA (siNC) were chemically synthesized (GenePharma, Shanghai, China). The target cells were transfected with 50 nM siRNA or negative control RNA and the vitro cell migration and invasion assays were performed 48 h after the siRNA transfection. Additionally, total RNAs and protein were prepared for real-time reverse transcription–polymerase chain reaction (RT–PCR) and western blotting analysis.

### miR-195 inhibitor experiments

To further investigate the function of miR-195, the antisense inhibitor (miR-195 inhibitor) experiments were performed to see whether the reverse effects to over-expression could be demonstrated. 2′-*O*-methyl modified miR-195 inhibitor (named as miR-195-Inhi, 5′-GCCAAUAUUUCUGUGCUGCUA-3′) and NC inhibitor (named as Inhi-NC, 5′-CAGUACUUUUGUGUAGUACAA-3′) were used. The cells were co-transfected with either miR-195 mimics or NC oligos with miR-195 inhibitor or NC inhibitor. After 48 h of transfection, transwell assay was assessed. In order to identify miR-195 targeting Fra-1 accurately, we established two groups: siFRA-1 + miR-195-Inhi, siFRA-1 + Inhi -NC, and then the cell migration and invasion should be assessed. Besides, expression level of miR-195 was calculated by quantitative PCR. In addition, the Fra-1 expression was determined by western blotting.

### Fra-1 rescue experiments

The Fra-1 over-expression plasmid was obtained by inserting the human Fra-1 complementary DNA lacking the 3′-UTR into the pIRES2-eGFP (Clontech, USA, the over-expression clone of Fra-1 would be termed as pFra-1) between the *Hin*dIII and *Xho*I sites. The vector was verified by sequencing before application. After preparation, miR-195 or miR-NC was co-transfected with pFra-1 or the empty control vector (pNull). The cells were collected and analyzed for migration and invasion as described above. The efficiency of Fra-1 over-expression was verified by western blotting.

### Tissue microarrays

A commercial human prostate cancer tissue microarray bearing 29 pairs of prostate cancer and corresponding non-tumor tissues was purchased from Shanghai Outdo Biotech (product ID: OD-CT-UrPrt03) for Fra-1 expression pattern analysis. For immunohistochemical (IHC) staining, tissue sections were deparaffinized in xylene, hydrated with graded ethanol and quenched for endogenous peroxidase activity in 0.3 % hydrogen peroxide. Microwave heating was performed in sodium citrate solution (pH 6.0) for epitope retrieval, and goat serum was used for blocking. The slides were incubated with anti-Fra-1 primary antibody (Epitomics, Burlingame, USA) at 4 °C for 16 h and incubated with a HRP-conjugated secondary antibody at room temperature for 1 h. DAB was applied for color development, dark brown was considered positive staining. Hematoxylin was applied for counterstain. The images were obtained under a microscope (Olympus, Japan). The Strength of positivity was semi-quantified by comprehensively considering staining intensity and the proportion of positive cells.

### Statistics

The data were expressed as the mean ± SD of three independent experiments. The statistical analysis were performed using SPSS 17.0 statistical software (SPSS, Chicago, IL, USA). Student’s *t* test and Two-way ANOVA were used to compare intergroup differences. A p value of <0.05 was considered to be statistically significant.

## Results

### The expression of miR-195 was frequently downregulated in human prostate cancer

**Fig. 1:**
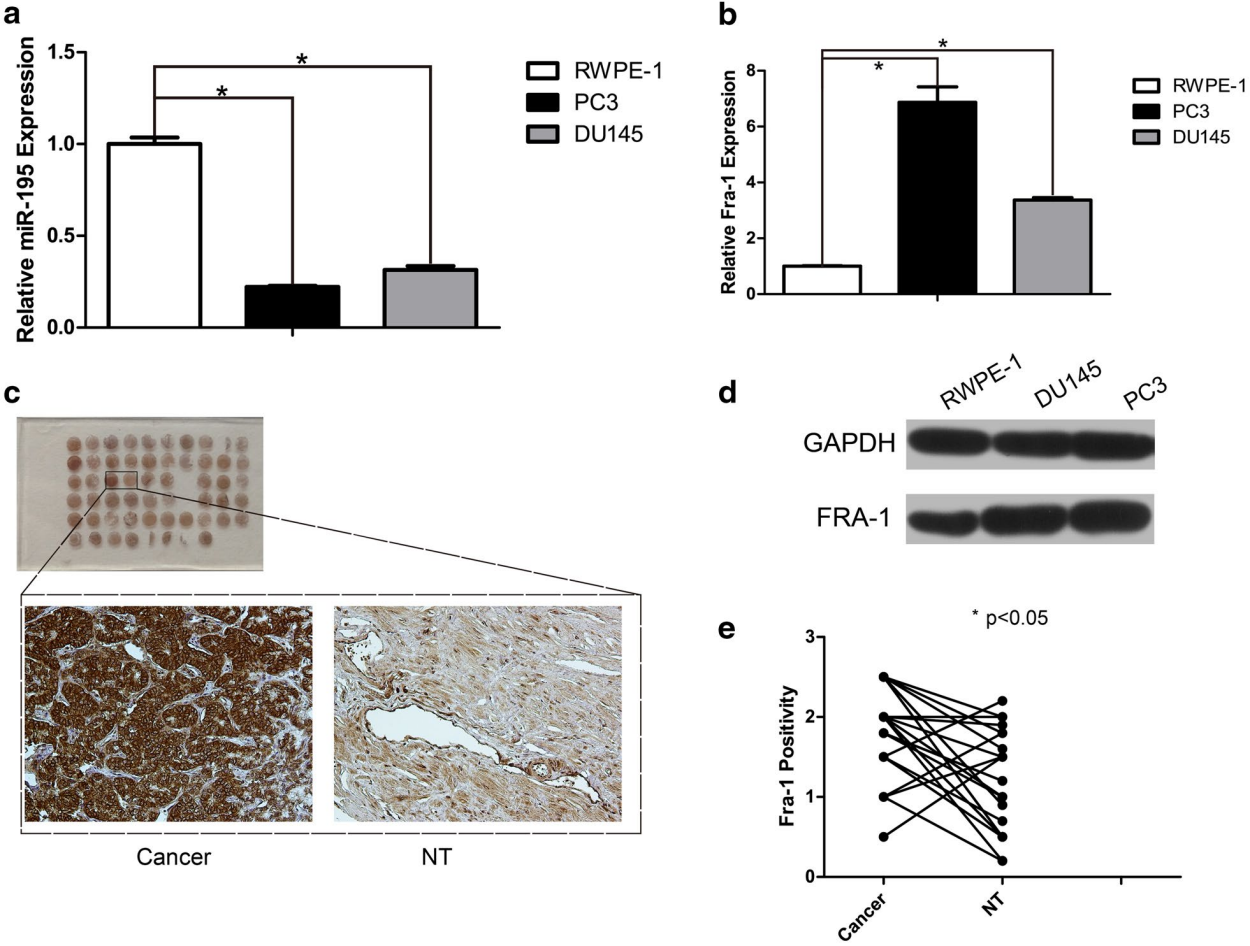
Quantitative analysis of miR-195 and Fra-1 in prostate cancer cell lines, IHC staining of Fra-1 expression pattern in tissue microarray. **a** The miR-195 levels in prostate cancer cell lines DU145 and PC3 were determined and compared with non-tumor prostate cell line RWPE-1. The real-time PCR analysis were normalized with U6 snRNA. **b** An increased expression pattern of Fra-1 in mRNA level was observed in DU145 and PC3 cells compared with RWPE-1 cells. **c** There were 29 pairs of tissue in tissue microarray, and three pairs of tissue were excluded from further semiquantitative analysis, since the corresponding tissue was lost during the staining process. IHC staining of Fra-1 in array and representative pair of dots was presented. **d** An increased protein expression of Fra-1 in DU145 and PC3 cells compared with RWPE-1. **e** Fra-1 showed in stronger positivity in 20 out of 29 cancer tissues (cancer) compared with the paired non-tumor tissues (NT) (P < 0.05)

Previous studies demonstrated that miR-195 was downregulated in prostate cancer [7], in this study, we examined the expression levels of miR-195 in one immortalized prostatic epithelial cell line, RWPE-1, and two prostate cancer cell lines, PC3 and DU145, by miR-quantitative RT-PCR analysis. As shown in Fig. 1a, prostate cancer cell lines had lower endogenous miR-195 levels when compared with the non-tumor epithelial cell line. Thus, we speculated that miR-195 might be a putative tumor suppressor in prostate cancer. In order to identify downstream targets of miR-195, bioinformatics analysis was carried out using online algorithms including TargetScan (http://targetscan.org/) and PicTar (http://pictar.mdc-berlin.de/cgi-bin/new_PicTar_vertebrate.cgi). We found that Fra-1 was a possible target of miR-195. Then the mRNA levels of Fra-1 in above three prostate cell lines were determined by quantitative PCR. An increased expression pattern of Fra-1 was observed in DU145 and PC3 cells compared with RWPE-1 cells (Fig. 1b, d). Furthermore, the expression levels of Fra-1 protein were markedly higher in cancerous tissues comparing with their non-cancerous counterparts in tissue microarray by IHC staining (Fig. 1e).Typical immunohistochemical findings of Fra-1 are shown in Fig. 1c. Detailed clinical information about this microarray was provided in Additional file 1: Table S1. These results indicated that high miR-195 level in normal prostatic epithelium cells might play a tumor-suppressive role through negatively regulating Fra-1 expression suggesting that downregulation of miR-195 might be involved in the prostate tumorigenesis and progression. Subsequently, we focused on the correlation between Fra-1 protein and miR-195.

### Introduction of miR-195 inhibited migration and invasion of prostate cancer cells in vitro

**Fig. 2:**
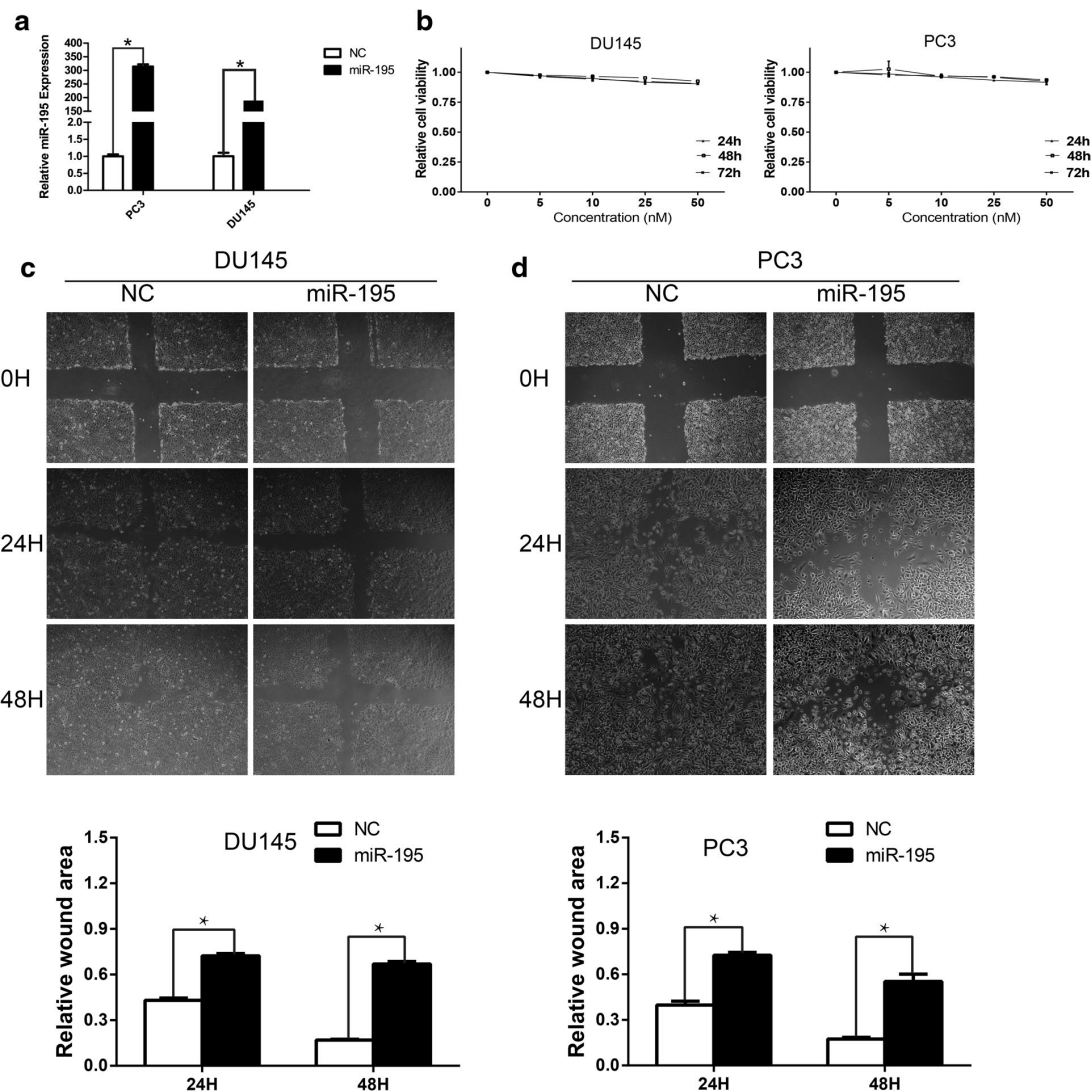
Forced expression of miR-195 suppressed cell motility in wound healing assay without significantly affecting cell viability. **a** The ectopic expression of miR-195 was confirmed by qRT-PCR. Three independent experiments were performed in each group. **b** Cell viability assay. Overexpression of miR-195 had no apparent effect on prostate cancer cell viability (cell viability of 0 nM was regarded as 1.0, n = 3). **c**, **d** DU145 and PC3 cells were transfected with NC, miR-195 mimics at a concentration of 50 nM and were performed wound healing assays with a 24–48 h recovery period, the wound area was measured by Image J software (*P < 0.05)

**Fig. 3:**
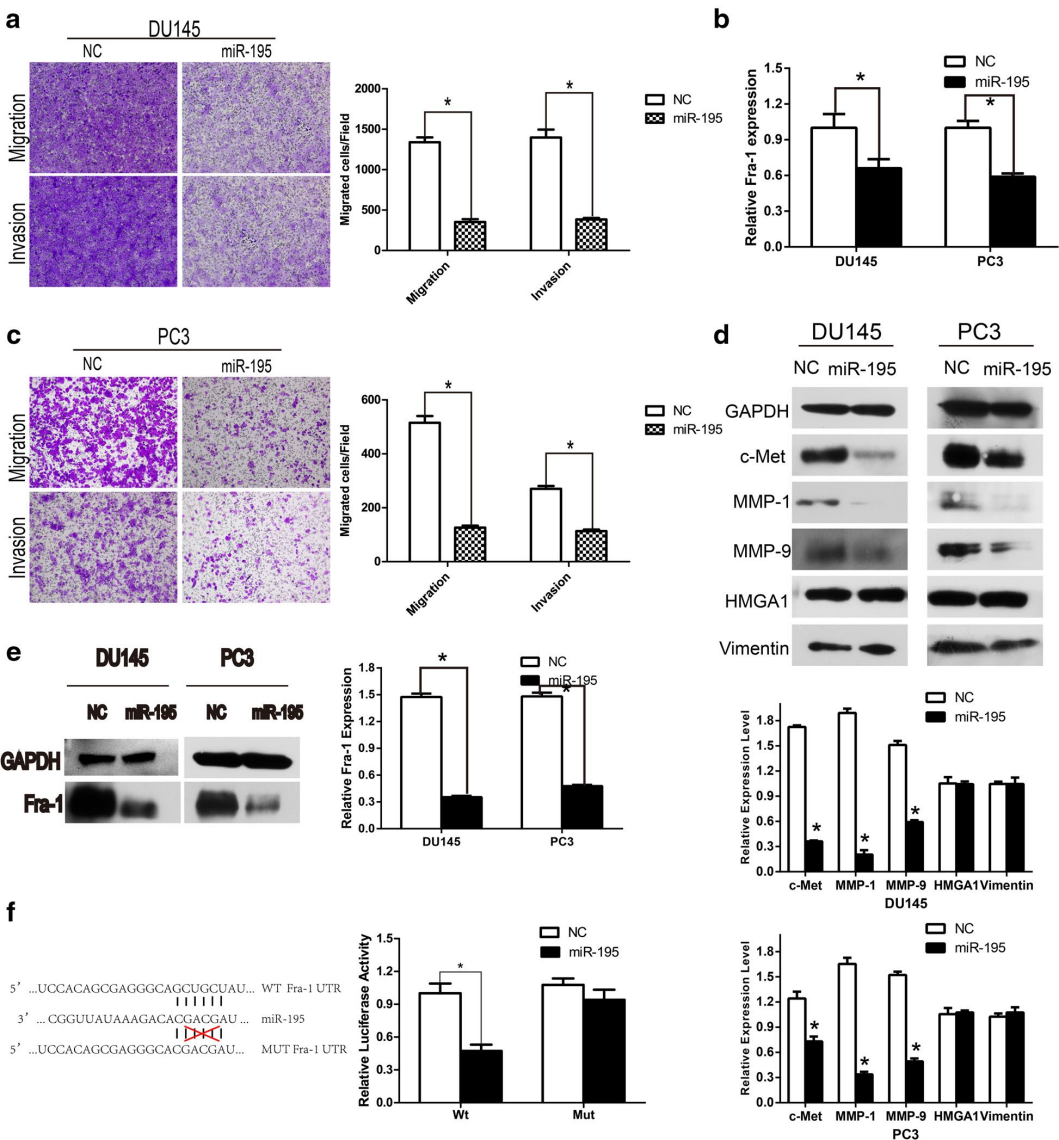
Transfection with miR-195 impaired the motility of prostate cancer cells and Fra-1 is a direct target of miR-195. **a**, **c** DU145 and PC3 cells were transfected with NC, miR-195 mimics and cell migration and invasion were assessed after 48 h incubation by transwell assay. Representative micrographs of the cell migration and invasion assay (*left*) and quantification (*right*) from three independent experiments were presented at ×100 (*P < 0.05). **b**, **e** The Fra-1 expression level was significantly reduced following treatment with miR-195 mimics by RT-PCR and western blot in DU145 and PC3 cells. **d** Representative western blotting results of motility-related proteins in miR-195-transfected DU145 and PC3 cells, band intensity quantification was made by Image J software, while GAPDH used as a loading control (n = 3; *P < 0.05). **f** A schematic of the computational predicted seed region in the 3′-UTR of Fra-1 (conserved in human, rhesus, dog, rat, mouse) was shown, while mutations on the “seed” sequences were designed as below. HEK293T cells were co-transfected with 50 nM of either miR-195 or NC and 200 ng pmirGLO Dual-Luciferase miRNA Target Expression Vector comprising Wt or Mut 3′-UTR of Fra-1. miR-195 significantly suppressed the firefly luciferase activity of construct with Wt 3′-UTR of Fra-1 (*P < 0.05)

To elucidate that whether miR-195 could function as a tumor suppressor, the effects of miR-195 over-expression was evaluated in vitro. First, we performed cell viability assay to investigate whether miR-195 has a biological function in proliferation of cancer cells, miR-195 mimics and negative control mimics at a concentration of 50 nM were separately transfected into both DU145 and PC3 cells. As shown in Fig. 2a, the ectopic expression of miR-195 was confirmed by qRT-PCR, and no significant difference was observed between NC group and miR-195 treated group, miR-195 did not significantly affect cell viability (Fig. 2b). However, forced expression of miR-195 in both cells led to retarded wound closing compared to NC groups (Fig. 2c, d) with cell wound healing assay. To further confirm the above findings, we decided to perform cell migration and invasion assays by using transwell chambers within 48 h after miRNA transfection. Transwell assay showed that the motility were significantly reduced in the miR-195 overexpressing PC3 and DU145 cells as compared with the negative control (Fig. 3a, c). Moreover, RWPE-1 was chosen to be an additional control and transfected by miR-195 and NC as mentioned above, the transwell assays were performed subsequently. The data was shown in additional file 2: Figure S1, which provide evidence to conclude that miR-195 indeed suppresses cell motility. Taken together, these results revealed that miR-195 negatively modulate prostate cancer cells migration and invasion.

### miR-195 inhibited Fra-1 expression

To verify that miR-195 act as a Fra-1 suppressor, PC-3 and DU145 cells were respectively transfected with 50 nM miR-195 and NC. Western blotting showed that at 72 h post-transfection, the overexpressions of miR-195 contributed to both significant decreases in Fra-1 protein levels in above two cell lines (Fig. 3e). On the other hand, qRT-PCR analysis demonstrated that Fra-1 mRNA levels had notable changes (Fig. 3b), suggesting that microRNA may suppress target gene expression through binding to its 3′-UTR.

### Fra-1 is a novel direct target of miR-195

As previously described, two algorithm programs were used to predict miRNAs targeting the 3′-UTR of Fra-1. As shown in Fig. 3f, Fra-1 mRNA has one potential complimentary miR-195 binding site within its 3′-UTR region. We next investigated whether Fra-1 was a direct functional target of miR-195. The 3′-UTR of Fra-1 was cloned into down-stream of firefly luciferase coding region of pmirGLO Dual-Luciferase miRNA Target Expression Vector. Additional vector with mutated putative binding sites was also constructed. Then HEK293T cells were co-transfected with either miR-195 or NC and reporter vectors containing wildtype (Wt) or mutant (Mut) 3′-UTR. The level of relative luciferase activity in the case of co-transfection with Wt 3′-UTR-reporter and miR-195 mimics was significantly lower than that co-transfected with NC. On the contrary, the level of luciferase activity in the reporter bearing 3′-UTR with mutated binding sites was unaffected by a simultaneous transfection with miR-195. These findings indicated that miR-195 inhibited Fra-1 expression through direct binding of 3′-UTR of its transcript. As illustrated in Fig. 3d, expression of motility-related markers, including c-Met, MMP1, MMP9, Vimentin and HMGA1 were compared by western blot between NC and miR-195 groups. The protein levels of these genes were significantly decreased in prostate cancer cells transfected with miR-195, except for vimentin and HMGA1.

### Decreased Fra-1 expression reduced the migration and invasion capability of prostate cancer cells

**Fig. 4:**
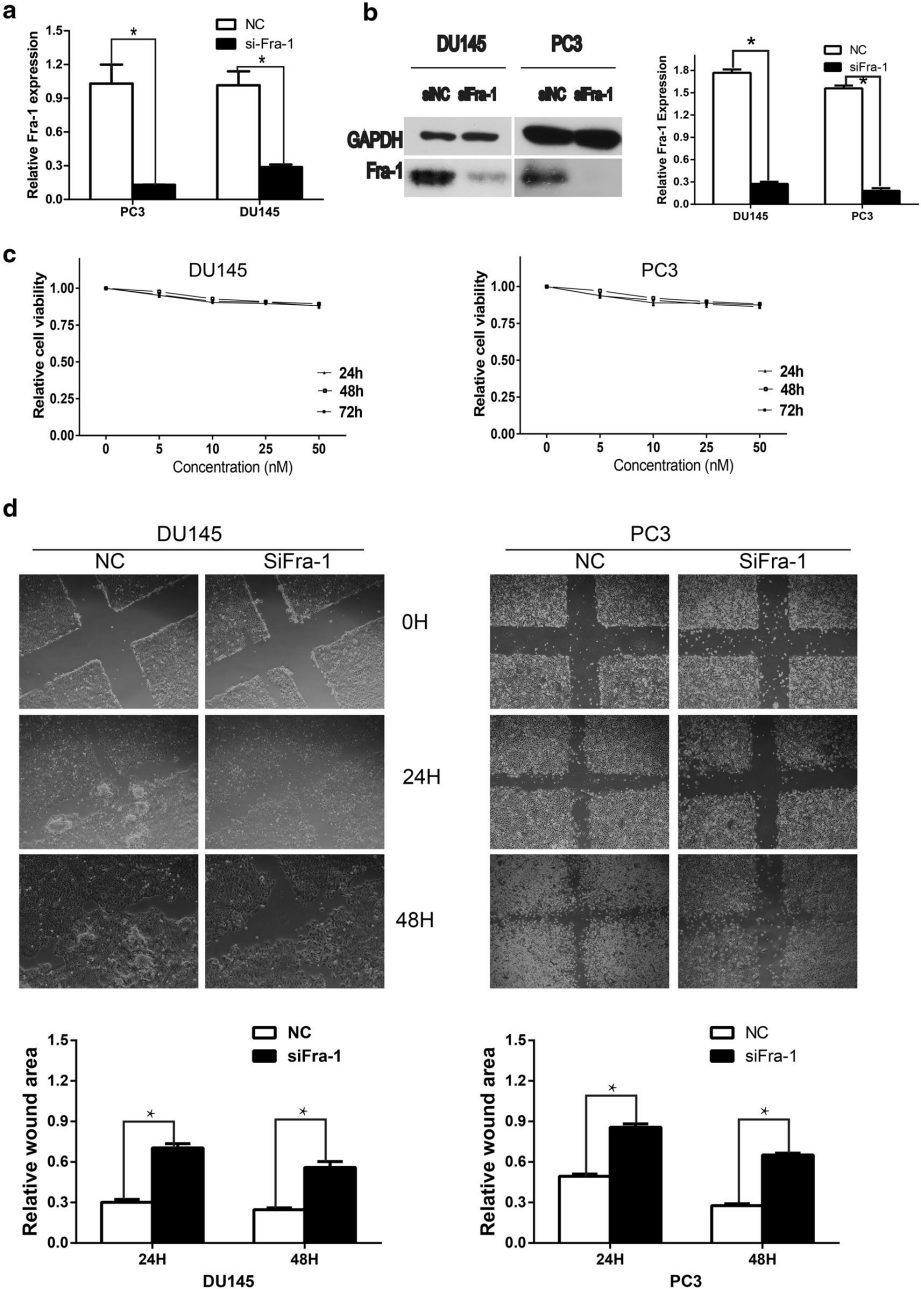
Silence of Fra-1 impaired cell motility in wound healing assay without significantly affecting cell viability. **a** The efficiency of siRNA was confirmed by real-time PCR. The relative Fra-1 mRNA levels were determined by RT-PCR in siRNA transfected cells (n = 3; *P < 0.05). **b** The knock-down of Fra-1 via RNAi technique reduced the expression of Fra-1 at protein level, while GAPDH used as a loading control (n = 3; *P < 0.05). **c** Transfection of siFra-1 had no apparent effect on prostate cancer cell viability, concentration of the reagent and time course of the cell proliferation (24, 48 and 72 h after transfection) were provided. **d** Wound healing assays with siFra-1 in DU145 and PC3 cells were performed at 24 and 48 h, the wound area was measured by Image J software (*P < 0.05)

**Fig. 5:**
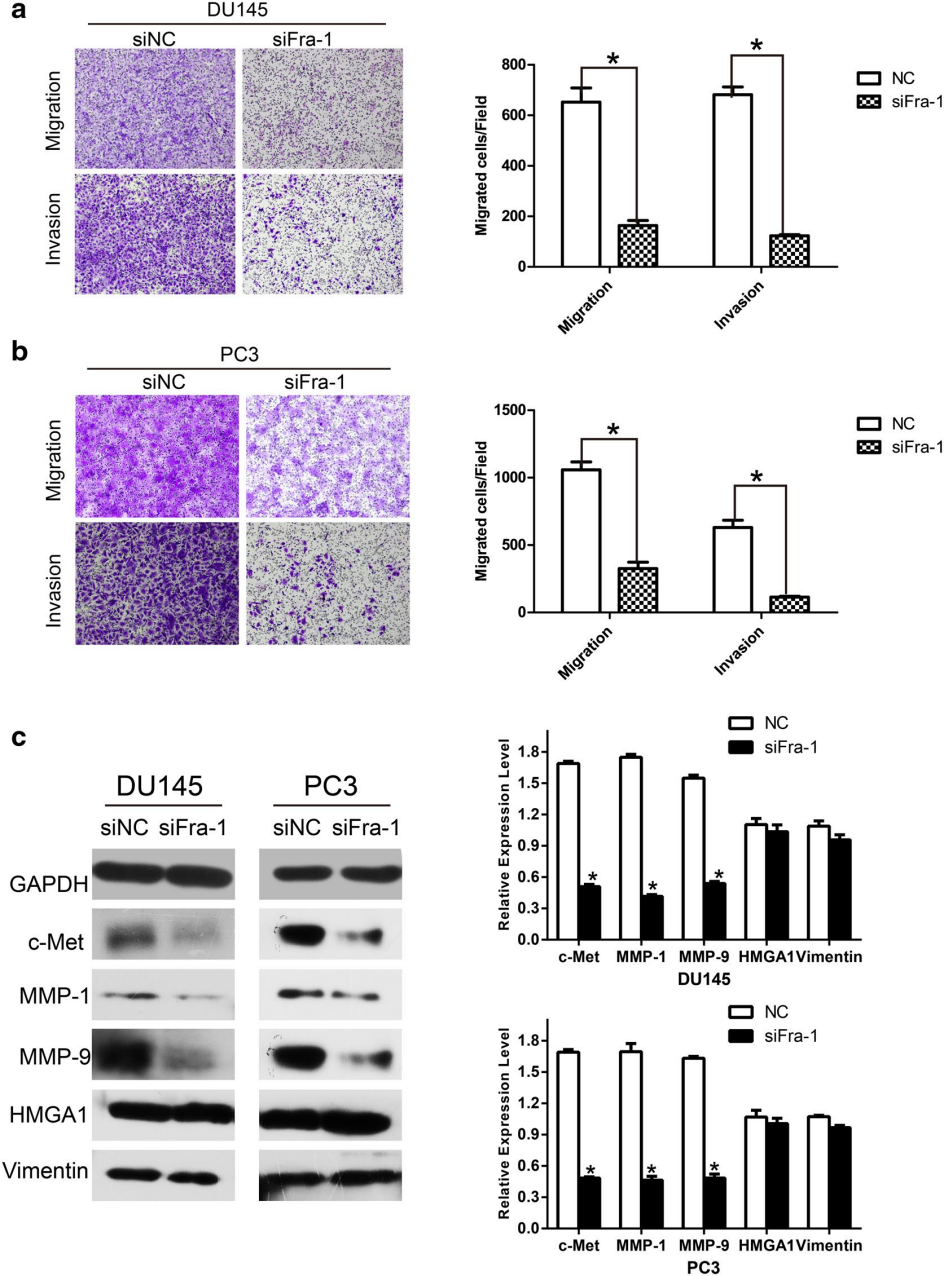
Knock-down of Fra-1 phenocopied the effect of miR-195. **a**, **b** siFra-1 yield an inhibitory effect on migration and invasion in both prostate cancer cell lines (×100), quantitative analysis were made from triplicate experiments (*P < 0.05). **c** Expression of motility-related markers were compared by western blot between NC and siFra-1 groups, band intensity quantification was calculated by Image J software, GAPDH was used as a loading control (*P < 0.05)

As a novel target of miR-195, Fra-1 was reported to regulate cell motility and invasion in various malignant epithelial cells. To determine whether these cell characteristics are affected by Fra-1 in prostate cancer cells, we knocked down Fra-1 with siRNA in PC3 and DU145 cells. The Fra-1 mRNA level was analyzed using RT-PCR at 48 h after transfection, which was obviously decreased in siFra-1 transfected prostate cancer cells (Fig. 4a). Knockdown of Fra-1 protein expression was also confirmed by Western analysis (Fig. 4b). With the efficient knockdown of Fra-1, we performed cell viability assay, there was no significant difference in both cell lines between siFra-1 groups and NC groups (Fig. 4c). Wound healing assay and transwell assay showed that the migration and invasion of siFra-1-transfected cells were significantly reduced as compared with the control cells (Figs. 4d, 5a, b). The knock-down of Fra-1 through RNAi approach yielded the anticipated cell physiological function, which phenocopied the effect of miR-195 over-expression. Furthermore, Fra-1 had been shown to regulate the expression of the MMP family members in many cancer cells [22, 23]. It also affected other regulators of cell motility, including c-Met, vimentin and HMGA1 in malignant cells [24, 25]. As exhibited in 5C, the protein expressions of c-Met, MMP1, MMP9 were decreased in siRNA transfected prostate cancer cells.

### Inhibition of miR-195 partially reverses the overexpression of miR-195 induced effects

**Fig. 6:**
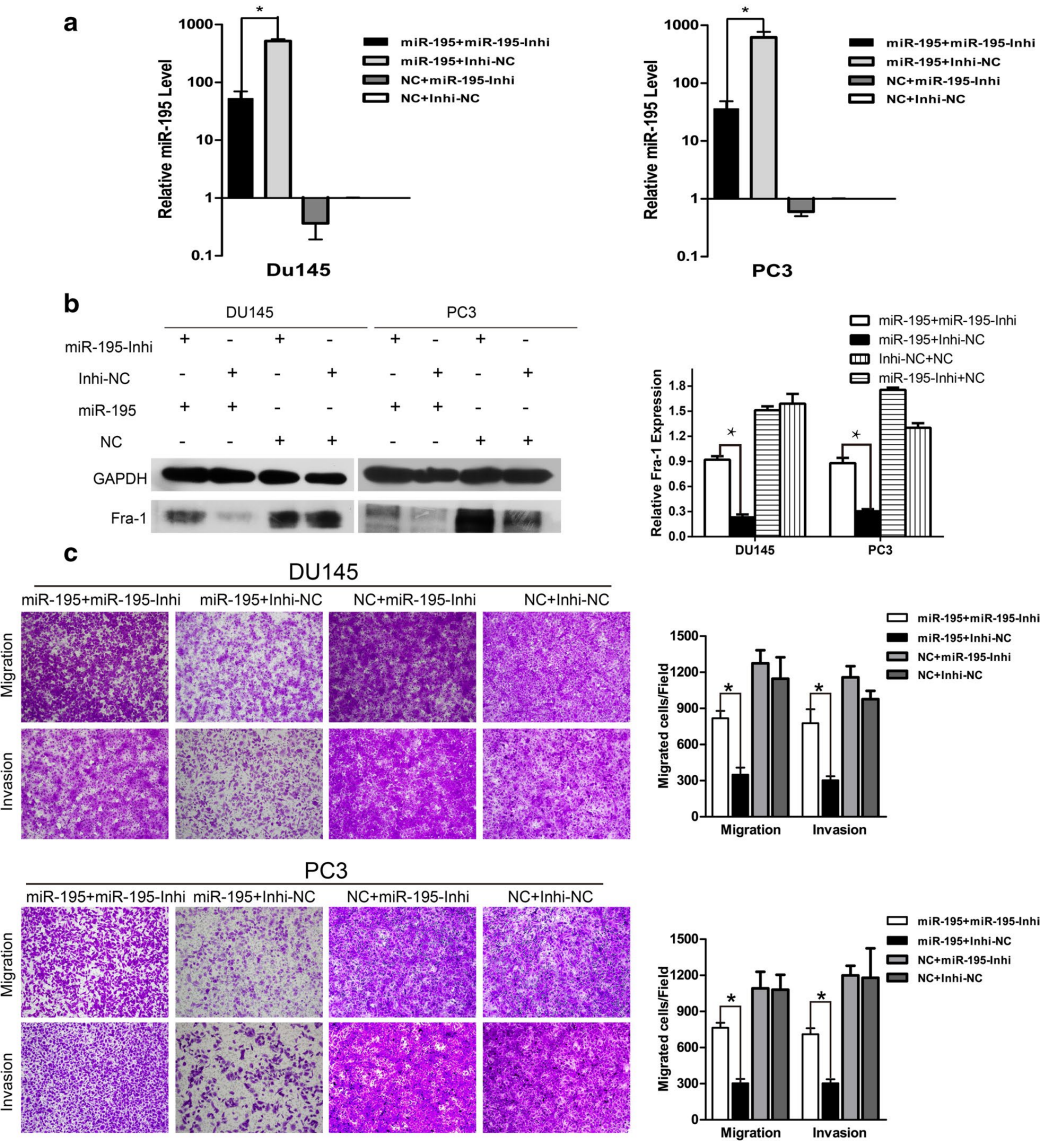
Inhibition of miR-195 partially reversed the over-expression of miR-195 induced effect. **a** Inhibitor of miR-195 suppressed the expression of miR-195. DU145 and PC3 cells were co-transfected with miR-195-Inhi (vs. Inhi-NC) and miR-195 (vs. NC). The expression of miR-195 was determined by real-time PCR, snRNA U6 served as an internal control (*P < 0.05). **b** The expression of Fra-1 was determined by western blot analysis after miR-195 inhibitor treatment, western-blot results should be analyzed quantitatively (*P < 0.05). **c** The prostate cancer cells migration and invasion ability was restored after miR-195-Inhi transfection (×100) (*P < 0.05)

**Fig. 7:**
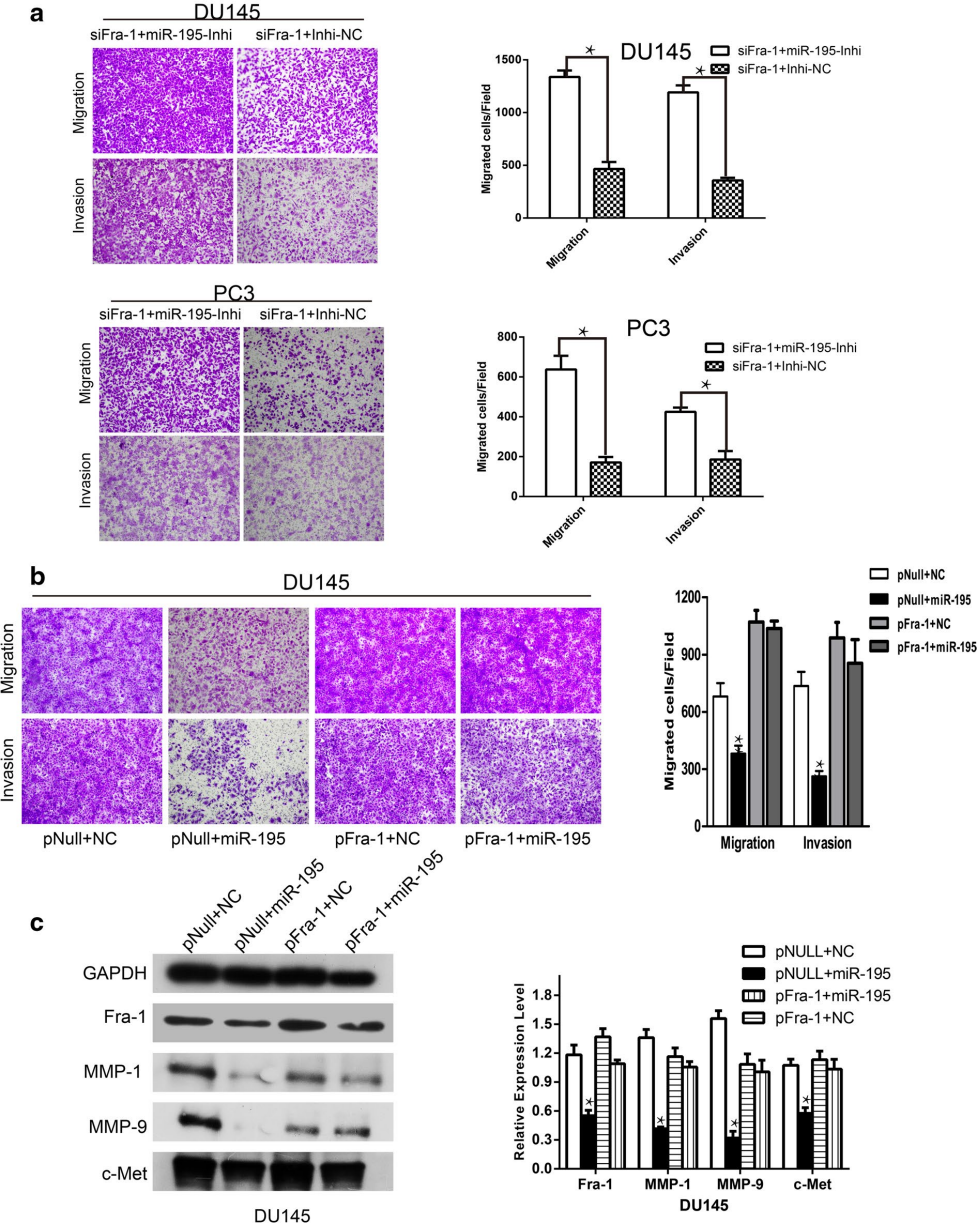
Forced expression of Fra-1 or miR-195-inhibitor partially rescued miR-195-dependent suppression of tumorous behavior. **a** DU145 and PC3 cells were co-transfected with either miR-195-inhibitor or Inhibitor-NC oligos with siFra-1. Transwell assays were conducted and quantified (*P < 0.05). **b** Forced expression of Fra-1 abrogated cell motility impairment by miR-195 (representative migration and invasion results at ×100 were shown). Quantitative analysis revealed a significant increase in cell migration and invasion after Fra-1 re-expression. **c** Western blot analysis was performed to confirm the re-expression of Fra-1 and other related proteins

To better verify the function of miR-195, the antisense inhibitor experiments were performed to see whether the reverse effects could be observed. As a result, co-transfection of miR-195-Inhi was applied to attenuate the miR-195 expression promotion in the level of mRNA (Fig. 6a). The Fra-1 expression inhibition by miR-195 in the level of protein could be reversed in both cells (Fig. 6b). Although PC3 and DU145 are both castration resistant prostate cancer cells, they have different genetic backgrounds and biological properties, which may respond to inhibitor oligos at different levels. Furthermore, miR-195-Inhi could partially abrogate the effect of miR-195 on cells migration and invasion ability (Fig. 6c). Moreover, the cell migration and invasion in cell lines with silence of Fra-1 could be partially restored when the miR-195 level was downregulated (Fig. 7a). Thus, we confirmed that miR-195-Inhi could reverse the effects to over-expression of miR-195.

### Forced Fra-1 expression partially rescued the migration and invasion capability of miR-195-transfected prostate cancer cells

To further investigate whether the effects of miR-195 on cell motility are mediated by Fra-1, we next carried out cell migration and invasion assays in DU145 cells that were co-transfected with miR-195 and the pFra-1 plasmid, which lacked the 3′-UTR of Fra-1 and therefore cannot be inhibited by miR-195. The results showed that forced Fra-1 expression increased the migratory and invasive capabilities of prostate cancer cells. More importantly, this restoration of Fra-1, partially, but significantly, rescued the migration and invasion capability of miR-195-transfected cells (Fig. 7b). Besides, the Fra-1, c-Met, MMP1, MMP9 protein expressions confirmed by western blot analysis were consistent with transwell assay (Fig. 7c). Taken together, these data suggested that the effects of miR-195 on cell migration and invasion were in part mediated by Fra-1.

## Discussion

It is well known that invasion and metastasis are the most significant biological characteristics of malignant tumors and advanced metastatic PCa is currently incurable. However, it is the great challenge of current basic and clinical research to identify novel molecular targets that could improve the therapeutic strategies to combat this lethal cancer, the application and efficacy of these therapies are still limited. During the last decades, an increasing number of studies have demonstrated that deregulation of miRNAs was a common event in tumor tissues and miRNAs would be treated as ideal tumor biomarkers or therapeutic targets [26–29]. Previously a systematic review has summarized that the deregulated miRNAs are common in prostate cancer [30]. Although a series of relevant studies have created signatures for prostate cancer, which will help further establish molecular diagnosis, prognosis and therapy using miRNAs, the function and mechanisms of specific miRNAs in prostate cancer are still poorly understood.

Recently, a number of studies have shown that the roles of miR-195 may be controversial in different types of cancers. miR-195 expression was reported to be elevated in chronic lymphocytic leukemia [31] and it was considered to be an oncomiR. However, in majority of cancers, miR-195 was suggested to function as a tumor suppressor in cancer development and progression, which was consistent with its expression patterns in hepatocellular carcinoma [19], adrenocortical carcinoma [32, 33], colorectal cancer [21], breast cancer [34], bladder cancer [35, 36] and squamous cell cancer of tongue [37]. More importantly, a miRNA signature study had also shown that miR-195 was down-regulated in prostate cancer [7]. Our previous work confirmed that miR-195 was frequently down-regulated in bladder cancer. miR-195, as a tumor suppressor in bladder cancer cells, could induce G1-phase arrest by targeting the novel target CDK4. But the underlying mechanisms mediating miR-195 deregulation in cancer development are still elusive, particularly in tumor metastasis, and whether the deregulation of miR-195 expression in cancer is mediated by epigenetic alterations remains to be further investigated. Therefore, the potential function of miR-195, such as suppressing cell migration and invasion in prostate cancer, triggers our initial interest.

In the current study, we revealed a decrease in expression of the newly identified tumor suppressive miR-195 in two human prostate cancer cell lines PC3 and DU145 compared with normal prostatic epithelial cells RWPE-1. Our quantification analysis yielded a similar expression pattern with former miRNA signature results. But whether the Fra-1 expression level inversely correlated with the miRNA expression profile in prostate cancer tissue needs to be further investigated.

Furthermore, we validated the functional roles of miR-195 in prostate cancer cell lines by gain of function study. By transfecting cancer cells with miR-195 mimics, we revealed that miR-195 was a potential metastasis suppressor for PCa as miR-195 expression significantly diminished the cell motility and invasion capability of PCa cells. A series of transwell experiments showed that the migration and invasion were significantly reduced in the selective miR-195 overexpressing PCa cells as compared with the control ones. Meanwhile, the downregulation of Fra-1 expression at both mRNA and protein levels was observed. Moreover, dual luciferase reporter assay confirmed that miR-195 could specifically inhibit Fra-1 by binding to a critical region located on its 3′-UTR. These results suggest a targeted downregulation of Fra-1 by miR-195 in prostate cancer, and miR-195 functions as an inhibitor of prostate cancer progression.

Fra-1 was frequently reported to be prominently associated with tumor invasiveness, metastatic dissemination. It was validated to be overexpressed in various human malignancies, including breast cancer [38, 39], lung adenocarcinoma [23] and colon carcinoma cells [25]. Consistent with these observations, our study showed higher expression of Fra-1 in 20 of 29 (69 %) tumor tissues as compared with the adjacent normal tissues in a prostate cancer tissue microarray. Despite accumulating evidence pointing to promotive role of Fra-1 in metastasis, its interaction with miR-195 in cell migration and invasion has never been investigated, and its precise molecular mechanisms in the cellular malignant and invasive phenotypes are not fully understood. Fra-1 was one of the transcription factor complex activator protein-1 (AP-1) which played a central role in regulating gene transcription in several biological processes, including cell proliferation, differentiation, transformation, inflammation, and pulmonary defense. AP-1 was constituted of a great variety of dimers composed of members of Jun (c-Jun, Jun-B and Jun-D) and Fos (c-Fos, Fos-B, Fra-1 and Fra-2) proto-oncogenes. Overexpression of Fra-1 was a common mechanism of constitutive AP-1 activation in tumors and associated with AP-1-mediated transformation [40]. Yet, more and more data presented support that a shift of Fra-1 expression is an important step in carcinogenesis and/or progression. Besides, it had been demonstrated that Fra-1 directly induced MMP-1 and MMP-9 promoter activities in breast cancer cells and stimulated MMP-2 and MMP-9 expression to enhance the motility and invasion of lung cancer cells [23]. More interestingly, inhibition of Fra-1 by miR-34a led to downregulation of MMP-1 and MMP-9 in colon cancer cells, and eventually inhibited cell migration and invasion [41]. Matrix metalloproteinases (MMPs), especially MMP1 and MMP9, are critical enzymes responsible for the degradation of the basement membrane (BM) and extracellular matrix (ECM), thereby contributing to cancer invasion and metastasis. Previous studies have indicated that MMP1 and MMP9 activity could be upregulated by c-Met [42]. Of note, in our study, MMP1, MMP9 and c-Met were also significantly decreased in miR-195-transfected cells, although they were not direct targets of miR-195. In addition, we also observed that MMP1, MMP9 and c-Met expression could be downregulated in Fra-1-silenced cells. These results indicated that miR-195 might reduce cell migration and invasion partially through indirectly regulating MMP1, MMP9 and c-Met. The above-mentioned reports may help to explain the mechanisms and pathways of anti-Fra-1 methods on migration and invasion of cancer cells.

Here, we found that the specific Fra-1 knock-down with siRNAs phenocopied the migration and invasion inhibitory effects of miR-195 over-expression, and forced Fra-1 expression by transfecting certain constructed plasmids or miR-195 inhibitor experiments could partially rescued the migration and invasion capability of miR-195-transfected cells, which further strengthened our findings that Fra-1 was an important mediator of miR-195-induced biological function.

## Conclusion

In conclusion, we were able to illustrate for the first time that miR-195 can regulate prostate cancer cell migration and invasion through its direct target gene Fra-1 by targeting it in a classical 3′-UTR binding fashion. Our findings suggest that miR-195 may function as a potential biological molecule for preventing metastasis of prostate cancer, which may lead more new diagnostic and therapeutic approaches for prostate cancer, and provide new insights into the posttranscriptional regulation of Fra-1. Indeed, it is probable that other regulators of Fra-1 may also participate in prostate cancer development and our future studies should pay more attention to examining how Fra-1 is regulated in PCa or other human diseases.

## Additional files

Additional file 1:
**Table S1. **Detailed clinical information of prostate cancer microarray. Additional file 2:
**Figure S1.** Transfection of miR-195 had no impact on motility of normal prostate cells (RWPE-1).
