# Supplementary material for: MicroRNA-195-5p, a new regulator of Fra-1, suppresses the migration and invasion of prostate cancer cells
Source: J Transl Med. 2015 Sep 4;13:289. doi: 10.1186/s12967-015-0650-6 (PMC4558968; doi:10.1186/s12967-015-0650-6)
Supplement: Additional file 1: — Table S1. Detailed clinical information of prostate cancer microarray. [file 12967_2015_650_MOESM1_ESM.doc]

Additional file : Table S1 Patients and tumor characteristics (n = 29)

|  | Age | Sex | Grade | Gleason |
| --- | --- | --- | --- | --- |
| 1 | 62 | male | II | 3+3=6 |
| 2 | 80 | male | II | 3+3=6 |
| 3 | 81 | male | III | 4+3=7 |
| 4 | 57 | male | II | 3+3=6 |
| 5 | 60 | male | III | 3+4=7 |
| 6 | 80 | male | II | 3+3=6 |
| 7 | 68 | male | II | 3+3=6 |
| 8 | 76 | male | III | 3+5=8 |
| 9 | 76 | male | III | 3+4=7 |
| 10 | 76 | male | II | 3+3=6 |
| 11 | 66 | male | II | 3+3=6 |
| 12 | 65 | male | II | 3+3=6 |
| 13 | 53 | male | II | 3+3=6 |
| 14 | 80 | male | II | 3+3=6 |
| 15 | 71 | male | II | 3+3=6 |
| 16 | 55 | male | II | 3+4=7 |
| 17 | 62 | male | III | 3+4=7 |
| 18 | 72 | male | II | 3+4=7 |
| 19 | 72 | male | III | 4+4=8 |
| 20 | 55 | male | II | 3+3=6 |
| 21 | 74 | male | II | 3+3=6 |
| 22 | 69 | male | III | 4+4=8 |
| 23 | 75 | male | II | 3+4=7 |
| 24 | 71 | male | II | 3+4=7 |
| 25 | 68 | male | II | 3+4=7 |
| 26 | 74 | male | II | 3+4=7 |
| 27 | 67 | male | II | 4+3=7 |
| 28 | 56 | male | II | 4+3=7 |
| 29 | 65 | male | II | 4+3=7 |
