# Supplementary figures and images for: MicroRNA-195-5p, a new regulator of Fra-1, suppresses the migration and invasion of prostate cancer cells
Source: J Transl Med. 2015 Sep 4;13:289. doi: 10.1186/s12967-015-0650-6 (PMC4558968; doi:10.1186/s12967-015-0650-6)

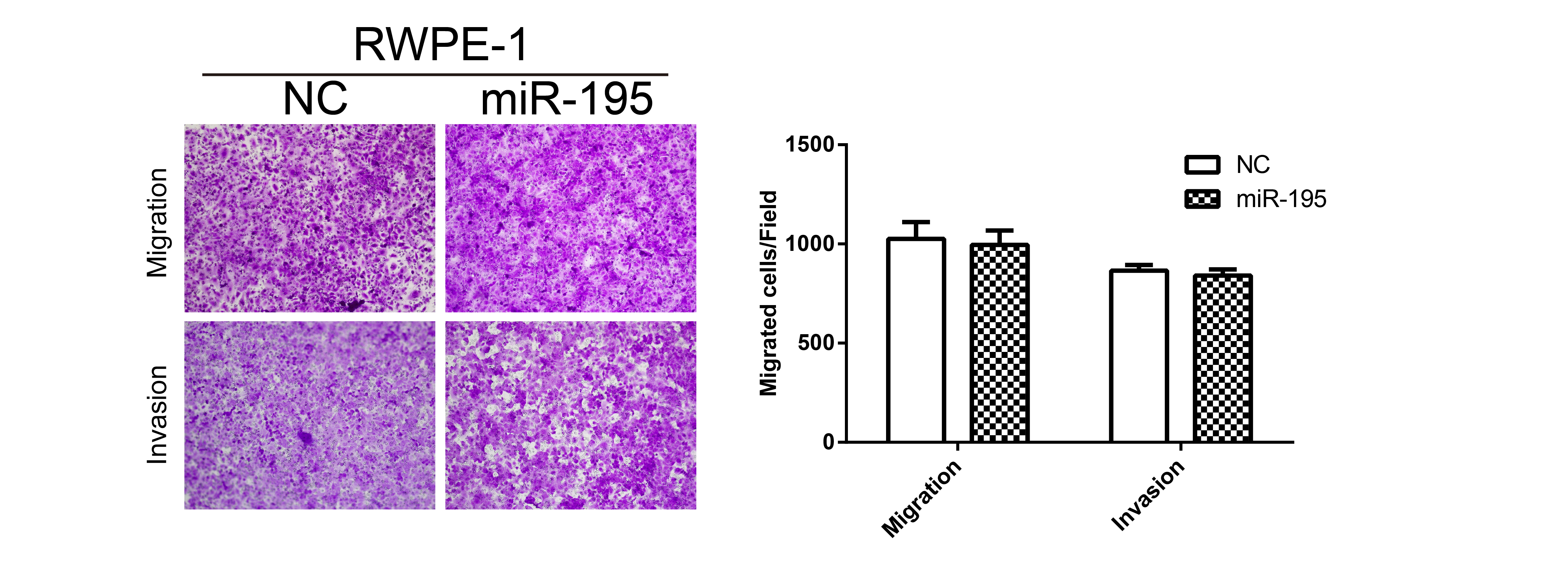

Supplement: Additional file 2: — Figure S1. Transfection of miR-195 had no impact on motility of normal prostate cells (RWPE-1). [file 12967_2015_650_MOESM2_ESM.tiff]
